# Chronic Hydrocephalus after Experimental Subarachnoid Hemorrhage

**DOI:** 10.1371/journal.pone.0069571

**Published:** 2013-07-23

**Authors:** Peter Lackner, Alexander Vahmjanin, Qin Hu, Paul R. Krafft, William Rolland, John H. Zhang

**Affiliations:** 1 Department of Physiology and Pharmacology, Loma Linda University, Loma Linda, California, United States of America; 2 Department of Neurology, Innsbruck Medical University, Innsbruck, Austria; University of Münster, Germany

## Abstract

Chronic communicating hydrocephalus is a significant health problem affecting up to 20% of survivors of spontaneous subarachnoid hemorrhage (SAH). The development of new treatment strategies is hampered by the lack of well characterized disease models. This study investigated the incidence of chronic hydrocephalus by evaluating the temporal profile of intracranial pressure (ICP) elevation after SAH, induced by endovascular perforation in rats. Twenty-five adult male Sprague-Dawley rats (260–320g) were subjected to either endovascular perforation or sham surgery. Five animals died after SAH induction. At 7, 14 and 21 days after surgery ICP was measured by stereotaxic puncture of the cisterna magna in SAH (n=10) and SHAM (n=10) animals. On day 21 T-maze test was performed and the number of alterations and latency to decision was recorded. On day 23, samples were processed for histological analyses. The relative ventricle area was evaluated in coronal Nissl stained sections. On day 7 after surgery all animals showed normal ICP. The absolute ICP values were significantly higher in SAH compared to SHAM animals on day 21 (8.26±4.53 mmHg versus 4.38±0.95 mmHg) but not on day 14. Observing an ICP of 10mmHg as cut-off, 3 animals showed elevated ICP on day 14 and another animal on day 21. The overall incidence of ICP elevation was 40% in SAH animals. On day 21, results of T-maze testing were significantly correlated with ICP values, i.e. animals with elevated ICP showed a lower number of alterations and a delayed decision. Histology yielded a significantly higher (3.59 fold increased) relative ventricle area in SAH animals with ICP elevation compared to SAH animals without ICP elevation. In conclusion, the current study shows that experimental SAH leads to chronic hydrocephalus, which is associated with ICP elevation, behavioral alterations and ventricular dilation in about 40% of SAH animals.

## Introduction

Aneurysmal subarachnoid hemorrhage (SAH) accounts for 5% of all strokes. In recent years mortality has decreased to about 10-20% due to improved interventional and surgical techniques allowing early aneurysm occlusion [1]. However, SAH is still associated with significant morbidity. Early brain injury and delayed cerebral ischemia cause neurological deficits and render about a third of the surviving patients unable to return to their previous work [1]. Another problem of the post-acute phase of the disease is chronic communicating hydrocephalus. This complication occurs in up to 20% of SAH survivors [2]. The consequences range from subtle neurocognitive and gait problems to severe disability requiring permanent cerebrospinal fluid (CSF) diversion. There is consensus that fibrosis of the leptomeninges and arachnoid granulations results from blood product deposition, which causes impaired CSF flow and decreased absorption [3]. Also partial obstruction of the fourth ventricular outflow and subsequent impaired CSF absorption may play a role [3]. However, no specific medical treatment for the prevention of chronic hydrocephalus is available. This is partly caused by the lack of appropriate models for SAH associated chronic hydrocephalus. One study evaluated the incidence of chronic hydrocephalus in experimental SAH. MRI and histology was applied to determine ventricle size and ventricular dilation was reported in about 40% of animals with SAH [4]. Yet, it is not clear if dilated ventricles are a result of chronic hydrocephalus or cortical atrophy secondary to ischemic events. Until now there is no long-term data on intracranial pressure in rodent models of SAH which could resolve this discrepancy.

Therefore, the current study was performed to evaluate the time course of ICP for 3 weeks after SAH, induced by endovascular perforation in rats, and to study its association with ventricular dilation and neurobehavioral deficits. 

## Materials and Methods

### Ethics Statement

This study was carried out in strict accordance with the recommendations in the Guide for the Care and Use of Laboratory Animals of the National Institutes of Health. All protocols were approved by the Institutional Animal Care and Use Committee of Loma Linda University. All surgery was performed under anesthesia with 3% isoflurane in 70%/30% medical-air/oxygen, animals received 25µg/kg Buprenorphine subcutaneously after surgery, and all efforts were made to minimize suffering.

### Animals and surgery

A total of 25 adult male Sprague-Dawley rats (Harlan, Indianapolis, IN) weighing 260–320 g were used for the study. The endovascular perforation model of SAH was performed as previously described [5,6]. Briefly, rats were anesthetized, intubated and kept on artificial ventilation during surgery. Body temperature was monitored by a rectal probe and normo-thermia was maintained by a heating lamp. A sharpened 4-0 nylon suture was introduced into the left internal carotid artery (ICA) until resistance was felt (approximately 18 mm from the common carotid bifurcation). The suture was then advanced until the resistance was overcome to perforate the bifurcation of the anterior and middle cerebral arteries. The suture was withdrawn immediately after perforation. In Sham operated animals the suture was inserted into the left ICA, however no vessel perforation was performed. After suture removal the incision was closed, and rats were individually housed in heated cages until recovery.

For ICP measurement intubated and artificially ventilated rats were mounted on a stereotaxic frame and the head was inclined to about 30 degrees. A midline skin incision was made and the atlanto-occipital membrane was exposed. The cisterna magna was stereotaxically punctured with a 26G Hamilton needle, which was connected to the pressure transducer of a Digi-Med LPA 400 – low pressure Analyzer (Micro-Med, Louisville, Kentucky, USA) via PE50 tubing. Correct placement of the needle in the subarachnoid space was confirmed if an immediate increase of ICP was observed after applying abdominal pressure. The puncture site was sealed with a drop of glue to prevent CSF leakage.

### Neurological scores

Twenty-four hours after the procedure modified Garcia score [7,8] and Beam balance test were performed. The modified Garcia score consists of six tests: spontaneous activity, symmetrical movements of limbs, forelimbs outstretching, climbing a wall of a wire cage, axillary touch response, and vibrissae touch response. The best score is 18 and the worst score is 2.

The beam balance test investigated the animal’s ability to walk on a narrow wooden beam (2.25cm diameter) for 60 seconds: four points, walking ≥20cm; three points, walking 10cm-20cm; two points, walking ≥10cm but falling; one point, walking <10cm; and zero points, falling with walking <10cm. The summative scores of three consecutive trials in a 5-minute interval were calculated.

Twenty-one days after SAH, the T-Maze test was performed. Rats were placed into the stem (40 cm × 10 cm) of a maze and allowed to explore until one arm (46 cm × 10 cm) was chosen. The latency to decision (seconds) was measured and the mean latency was calculated. From the sequence of eleven trials, of left and right arm choices, the rate of spontaneous alternation was evaluated [9,10]. Neurological scores were evaluated in a blinded fashion.

### Histology

Rats were fatally anesthetized with isoflurane (≥ % 5) followed by cardiovascular perfusion with ice-cold PBS and 10% formaldehyde. Brains were removed and postfixed/cryoprotected in 10% formaldehyde / 30% sucrose for 3 days, embedded in mounting medium and frozen in liquid nitrogen. For all neuropathological analyses 10μm thick coronal sections were cut on a cryostat (Leica Microsystems LM3050S), mounted on poly-L-lysine-coated slides and Nissl stained. Morphometric analysis of stained slides at bregma 0 (+/- 250µm) was performed by computer-assisted (Photoshop CS4, Adobe, San Jose, CA, USA) hand delineation of the ventricles, cortex and the total brain area. The relative ventricle area was calculated as ventricle area / total brain area. The relative cortex area was calculated as cortex area / (total brain area-ventricle area).

### Statistical analyses

Neurological scores and relative ventricle areas were compared between groups using Wilcoxon rank-sum test. ICP values were analyzed by repeated measures two-way ANOVA and post-hoc comparison of SHAM vs. SAH animals were Bonferroni-corrected. Correlations between ICP values and T-mace test results were assessed by Spearman’s rho. To evaluate the predictive value of baseline variables (modified Garcia score, beam walking test, ICP at day 7, change of body weight in the first 7 days) logistic regression models were calculated. Mean and standard deviation are given. All analyses were done using GraphPad Prism 5.00 software (GraphPad Prism Software Inc., San Diego, CA, USA).

## Results

**Figure 1:**
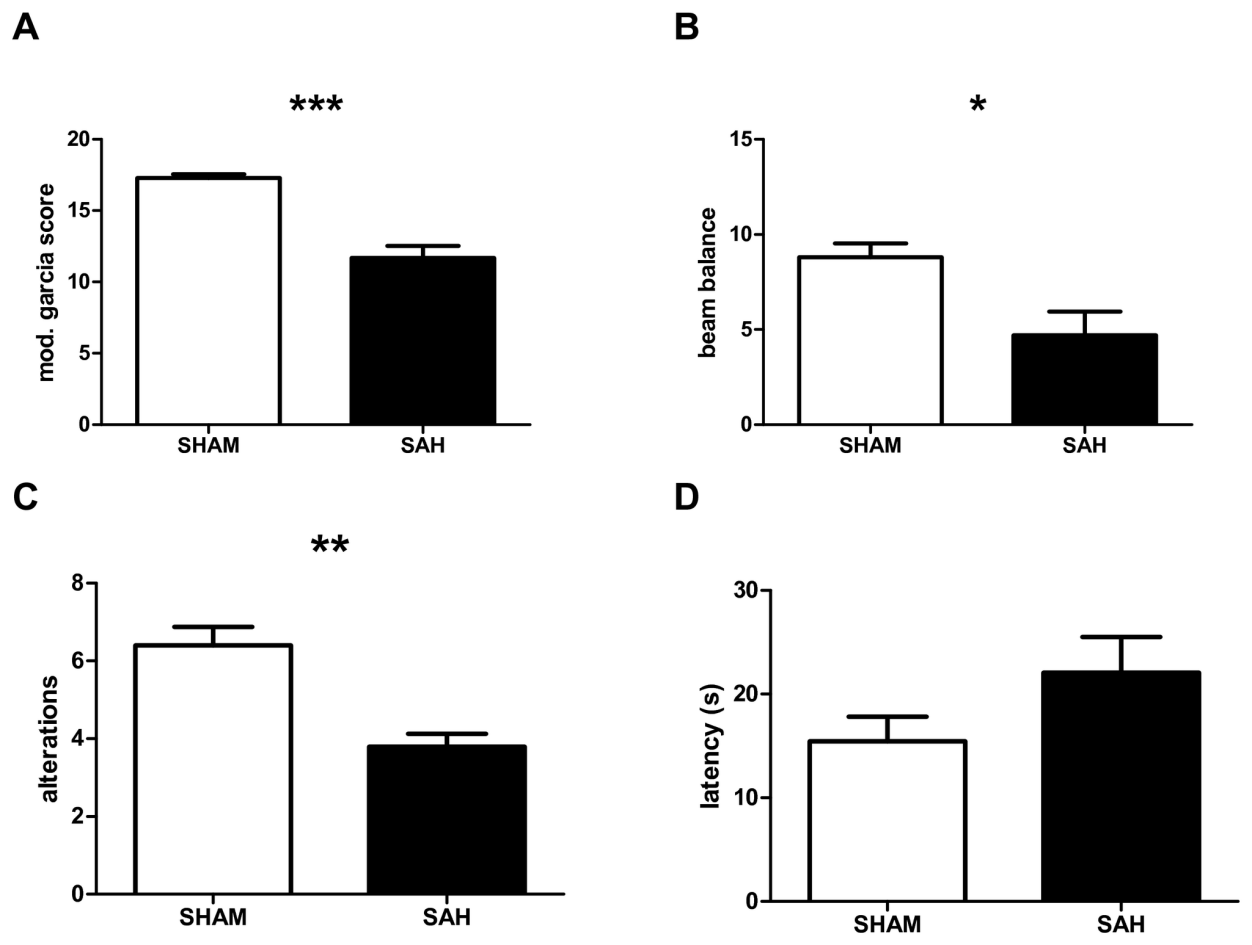
Neurobehavioral scores. Neurobehavioral scores 24 hours (A, B) and 21 days (C, D) after SHAM or SAH surgery respectively. Modified Garcia score (A), sum score of three consecutive trials of beam balance test (B), number of alterations in T-maze test (C) and mean latency to decision in seconds in T-maze test (D). Mean and SEM is shown. * p<0.05, ** p<0.01, *** p<0.001.

Fifteen animals were subjected to SAH and ten animals to SHAM surgery. No SHAM animals died. Five animals died in the first 24h after SAH induction, yielding a surgery-associated mortality of 33%. The remaining 20 animals (SAH n=10, SHAM n=10) were included in the ICP study. Modified Garcia score and beam walk test results were significantly lower 24h after surgery in SAH animals (p<0.001, p<0.05, Figure 1A-B). On day 21, SAH animals showed significantly fewer alterations on the T-maze test compared to SHAM animals (p<0.01, Figure 1C). There was no significant difference in the latency to decision between SAH and SHAM animals (Figure 1D).

### ICP and behavior

**Figure 2:**
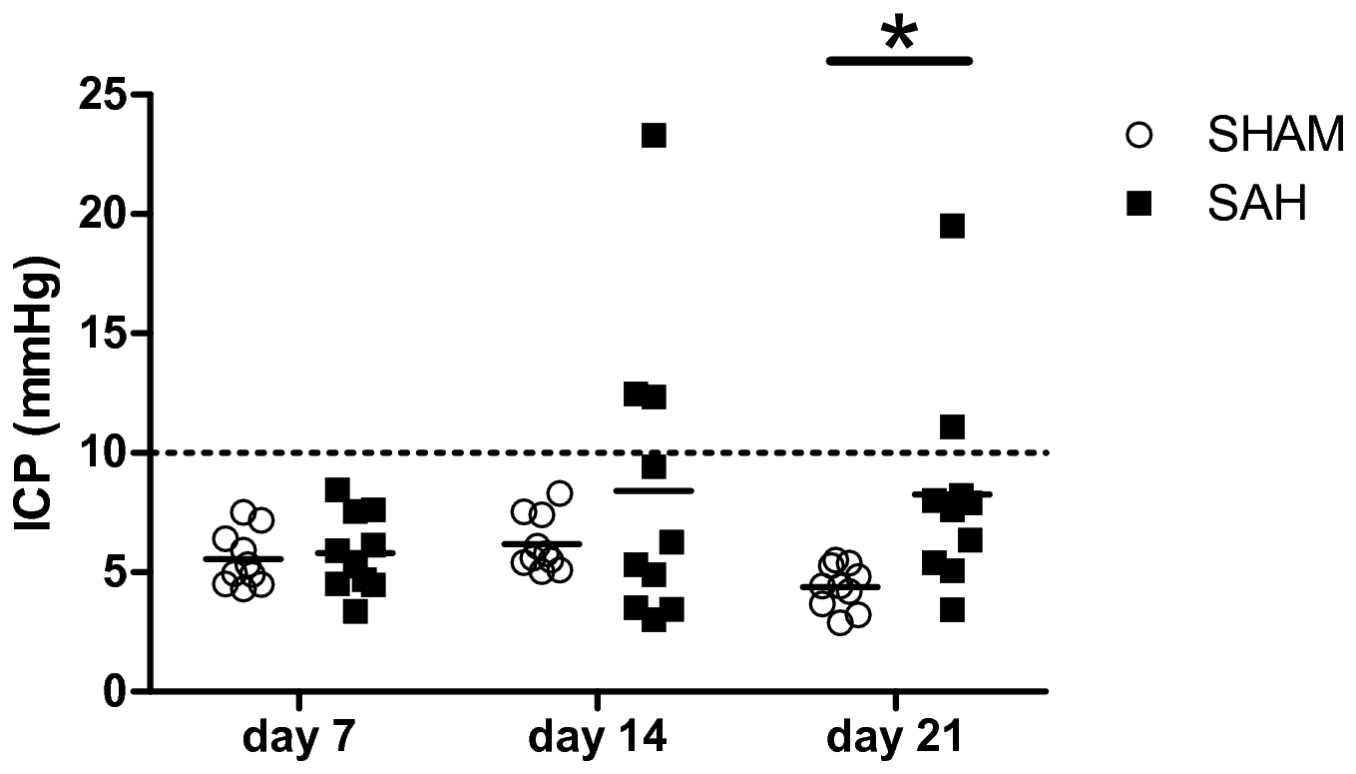
Time course of intracranial pressure. Intracranial pressure (ICP) in mmHg at three different time points in SHAM or SAH animals. Repeated-measures two-way ANOVA showed a significantly different time course of ICP in SHAM or SAH animals (p<0.05). * p<0.05 for the post-hoc comparison of SHAM vs. SAH at day 21 (Bonferroni-corrected).

**Figure 3:**
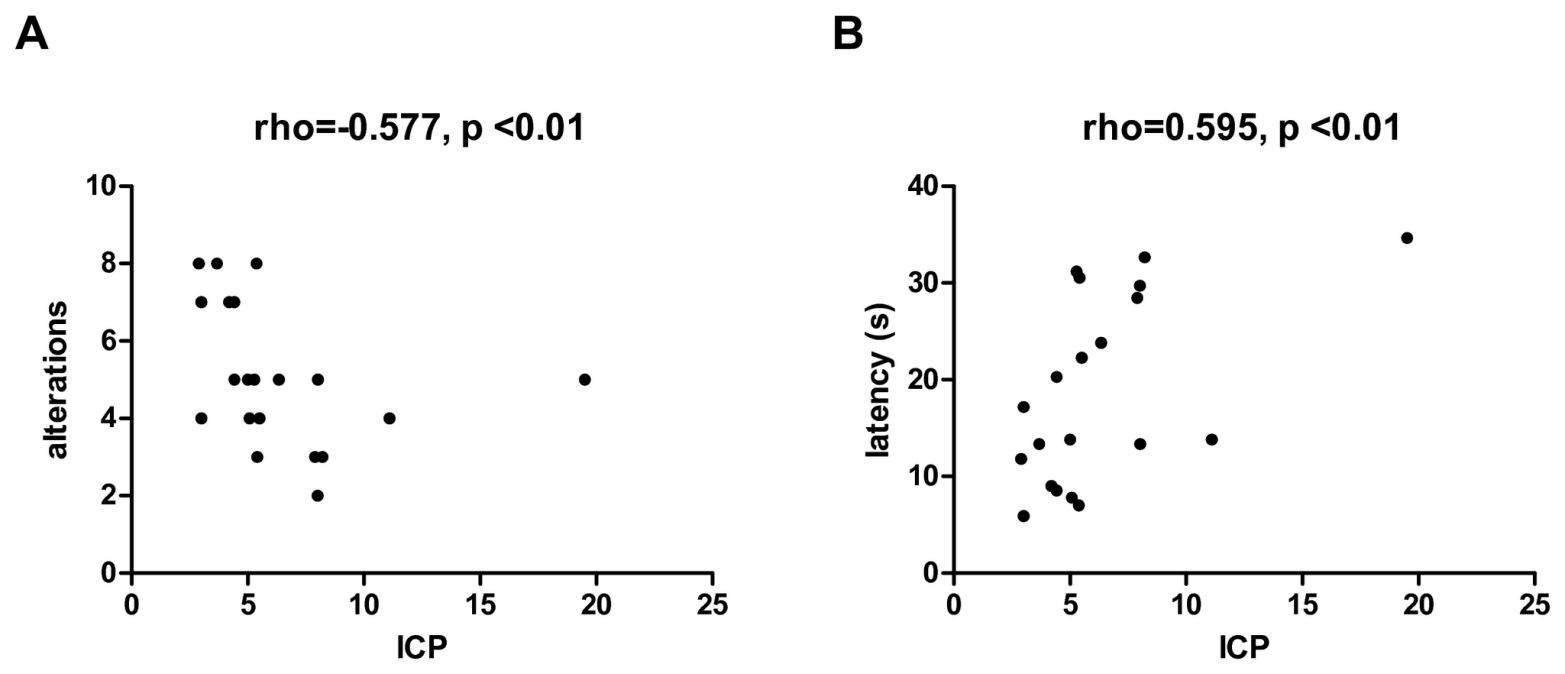
Correlation between behavior and intracranial pressure. Correlation analyses between the results of T-maze test at day 21 after surgery and intracranial pressure (ICP) in SHAM and SAH animals. Number of alterations (A) and mean latency to decision in seconds (B). Spearman’s rho and p-values are given.

At 7, 14 and 21 days after SAH or SHAM surgery animals were subjected to ICP measurements. At day 7 ICP values of SAH animals (5.79±1.75 mmHg) and SHAM animals (5.53±1.19 mmHg) were normal (Figure 2). At day 14 SAH animals showed increased ICP values (8.25±6.35 mmHg) while SHAM animals still had normal ICP values (6.13±1.19 mmHg). At day 21 ICP values in SAH animals (8.26±4.53 mmHg) were still increased compared to SHAM animals (4.38±0.95 mmHg). Repeated-measures two-way ANOVA showed a significantly different time course of ICP in SAH versus SHAM animals (p<0.05). Post-hoc comparisons yielded significantly higher ICP values in SAH animals at day 21 compared to SHAM animals (p<0.05, Figure 2). When observing an ICP value of 10 mmHg as cut-off, 3 animals showed elevated ICP on day 14 post-SAH. In two of these animals ICP decreased in the next measurement 21 days post SAH, one animal still had elevated ICP and another animal showed elevated ICP which had normal values on day 14. Therefore, the overall incidence of ICP elevation was 40%. In correlation analyses, a significant association between the results of T-maze test and ICP values on day 21 was observed. Hence, animals with elevated ICP showed less alterations and a higher latency to decision (Figure 3 A-B).

### ICP and histology

**Figure 4:**
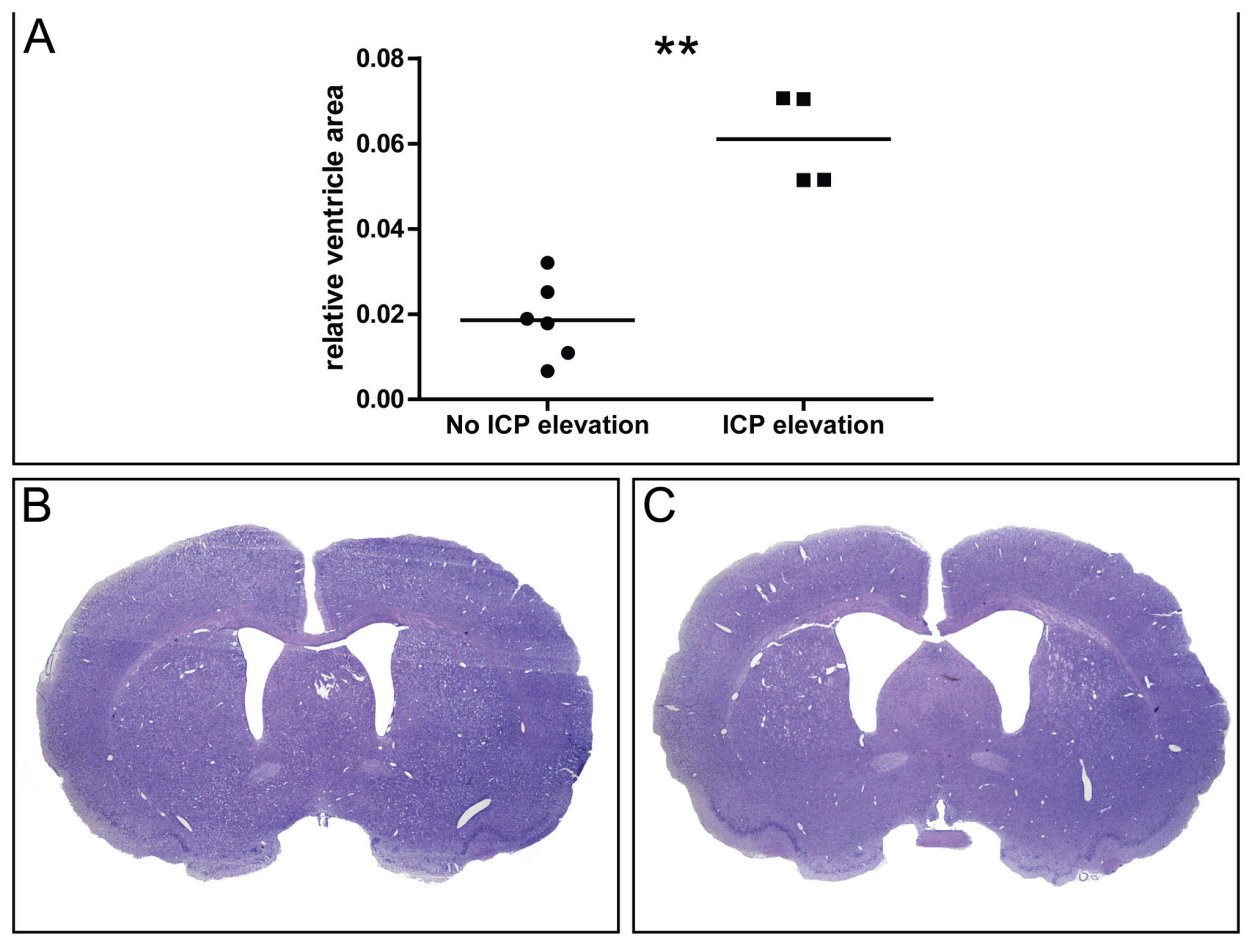
Intracranial pressure and histology. Relative ventricle area (ventricle area / brain area) (A) in SAH animals with normal ICP (≤ 10 mmHg) or elevated ICP (> 10 mmHg). Representative micrographs of coronal Nissl stained brain sections in a SAH animal with normal ICP (B) and a SAH animal with ICP elevation (C). Mean is shown. ** p<0.01.

At day 23 animals were killed and histology was performed. The mean relative ventricle size was 2.87±1.54% in SHAM animals versus 3.55±2.38% in SAH animals. This difference was not statistically significant (p=0.631). In contrast, SAH animals showing elevated ICP of more than 10 mmHg showed a highly significant enlargement of the relative ventricle size compared to SAH animals without ICP elevation (p<0.01, Figure 4A). The mean relative ventricle size in SAH animals without ICP elevation was 1.87±0.92% (Figure 4B) versus 6.1±1.09% (Figure 4C) in animals with elevated ICP, representing a 3.59 fold increase of the relative ventricle area. There was no significant difference of the relative area of the cortex between SAH animals with elevated or normal ICP (47.5±5.72% versus 47.3±3.78%) and there was no correlation between the relative cortex and ventricle area (rho = 0.01, p=0.965).

To evaluate the predictive value of baseline variables (modified Garcia score, beam walking test, ICP at day 7, change of body weight in the first 7 days) logistic regression models were calculated. No significant predictors for the development of ICP elevation or ventricular dilation could be identified.

## Discussion

This study provides a comprehensive analysis of the long term dynamics of intracranial pressure after subarachnoid hemorrhage induced by endovascular perforation in rats. The data shows that 40% of the animals develop ICP elevation which is associated with ventricular dilation — the typical characteristics of chronic communicating hydrocephalus. In addition, animals with elevated ICP showed behavioral deficits supporting its clinical relevance.

As of now, the endovascular perforation model is thought to be the most appropriate model to study early brain injury after SAH [11]. However, it has not been well characterized regarding the chronic effects of bleeding. Yet, there is some data about chronic hydrocephalus after experimental subarachnoid hemorrhage induced by injection of autologous blood into the cisterna magna. Using MRI and histology, ventricular dilation could be found in 42% of the rats [4]. This percentage is very similar with our results, in that 40% of the animals showed ICP elevation beginning 2 weeks after SAH induction. The important difference of our approach is the application of repeated ICP measurements as a marker for chronic hydrocephalus. It should be noted that in 60% of SAH animals ICP values were normal throughout the study and the relative ventricle area was not increased. Therefore the mean ICP value was only slightly increased in SAH compared to SHAM animals. In contrast, when observing 10 mmHg as a cut-off for elevated ICP [12] the relative ventricle area was significantly enlarged in SAH animals with ICP values above this cut-off. In addition the relative cortex area did not show any differences between the groups and there was no correlation with the relative ventricle area. These findings support the conclusion that ventricular dilation after experimental SAH is mainly a consequence of CSF diversion impairment and probably only to a lesser extend due to neuronal cell death and cortical atrophy.

The changes of brain physiology during the acute phase of SAH have been studied by various groups. Using the cisterna magna injection model in rats, ICP was measured for 5 days. Elevated ICP values were detected only in the first 24 hours after SAH induction [13]. Similar results were observed in a mouse endovascular perforation model, with elevated ICP 24 hours after SAH but normal values thereafter [14]. Recently, a MRI study in rats undergoing endovascular perforation provided evidence of acute occlusive hydrocephalus in 44% of rats after 24 hours [15]. These observations support the suitability of rodent models for this aspect of acute brain injury after SAH. Another study used a permanent cisterna magna cannula in rats undergoing SAH induction by transclival basilar artery puncture and was able to produce reliable pressure tracings for up 3 days after SAH [12]. This study reports similar baseline values as in our study with normal ICP below 10mmHg and a transient rise of ICP shortly after SAH induction. However, to our knowledge the current study is the first to investigate the long term course of ICP in a rodent model of SAH. We show that ICP values are normal 7 days after SAH. Animals show ICP elevations starting 14 days after SAH. Interestingly, in 2 animals with highly elevated ICP on day 14, ICP was decreased on day 21. This might be interpreted as a consequence of repeated puncture of the cisterna magna with putative CSF loss. In the current study the puncture site was sealed directly after the removal of the Hamilton needle similar to the reports of others [16,17]. Therefore it is unlikely that a significant CSF loss occurred. Despite the fact that ICP values decreased 21 days after SAH, animals with elevated ICP showed dilated ventricles consistent with communicating hydrocephalus. In a dog model of chronic hydrocephalus it has been shown that CSF pressure is initially increased, but when the ventricles become enlarged the pressure falls into the normal range again [18]. This could also be the case in the current study and would explain our observations. Further studies – using repeated MRI or micro CT - will have to clarify the time course of ICP elevation in relation to ventricular dilation. The results of T-maze test were correlated with ICP values, i.e. animals with higher ICP values showed a lower number of alterations and a higher latency to decision. T-maze test reflects working memory and latencies to decision have been shown to be delayed in animals with brain lesions [10]. One important aspect of chronic hydrocephalus in patients after SAH is progressive memory deficits and dementia. The observed correlation between ICP elevation and behavioral deficits indicate that the endovascular perforation model might be suitable to study chronic hydrocephalus. Since chronic hydrocephalus does not occur in all SAH animals after endovascular perforation, further studies aiming at increasing its rate are warranted to improve the applicability of this model for treatment studies. In addition, the long term effects on behavior and ventricular dilation remain to be resolved.

In conclusion, this study shows that ICP measurements by repeated puncture of the cisterna magna are feasible in rats after endovascular perforation. This method is able to detect chronic hydrocephalus — a condition associated with ICP elevations, ventricular dilation and behavioral deficits in about 40% of injured animals. The current model could be applied to study novel prophylactic therapies aiming at reducing the rate of patients requiring permanent CSF diversion after SAH.
